# Integrating Point of Care Ultrasound into Nephrology Fellowship Training: Insights from a Pilot Program

**DOI:** 10.24908/pocus.v7iKidney.14987

**Published:** 2022-02-01

**Authors:** Ann Young, Benoit Imbeault, Alberto Goffi, Alireza Zahirieh, Claire Kennedy, Daniel Blum, Ron Wald, William Beaubien-Souligny

**Affiliations:** 1 Division of Nephrology, St. Michael's Hospital Toronto Canada; 2 Division of Nephrology, UHN Toronto General Hospital Toronto Canada; 3 Division of Nephrology, Hopital Maisonneuve Rosemont Montréal Canada; 4 Interdepartmental Division of Critical Care Medicine, University of Toronto Toronto Canada; 5 Department of Critical Care Medicine, St. Michael's Hospital Toronto Canada; 6 Division of Nephrology, Sunnybrook Health Sciences Center Toronto Canada; 7 Division of Nephrology , Jewish General Hospital Montréal Canada; 8 Division of Nephrology, Centre Hospitalier de l'Université de Montréal Montréal Canada

**Keywords:** Point-of-Care ultrasound, medical education, imaging, renal ultrasound

 In nephrology, point of care ultrasound (POCUS) has multiple applications including the rapid evaluation of acute kidney injury, enhancing the initial evaluation of chronic kidney disease, direct evaluation of vascular access, and improved fluid balance management in acute and chronic settings 1, 2. Recently, the role of POCUS has been formally acknowledged by the American College of Physicians and curricula specific to nephrology have been proposed 3, 4. However, the integration of a novel clinical skill into a field comes with its unique set of challenges. Above all, most nephrologists in leadership roles within fellowship training programs lack POCUS experience, which represents a significant barrier for adequate exposure and teaching. Although educational curricula centered on nephrology have been proposed, the optimal model to ensure adequate POCUS exposure considering the scarcity of expertise among educators is not known. 

In 2018, we aimed to develop a pilot POCUS program for nephrology fellows at the University of Toronto. The focus was limited to renal POCUS with the aim of enabling the operator to detect hydronephrosis and to evaluate chronic kidney disease by measuring longitudinal kidney length. The objective was to provide adequate POCUS exposure without a dedicated POCUS rotation. During phase 1, the trainees were given a brief document demonstrating optimal transducer position for kidney assessment, kidney measurements, examples of hydronephrosis and renal atrophy. Two weeks later, a two-hour hands-on POCUS session was held. Six stations were organized with experienced instructors and volunteer models including healthy individuals and patients with renal anomalies including hydronephrosis and renal atrophy. Instructors included four nephrologists, one intensivist and one ultrasound technician with experience ranging from 1 to 15 years. Trainees had the opportunity to rotate between stations to benefit from various experience and teaching styles.

For phase 2, resources for self-directed learning were organized at two hospitals. These included access to dedicated POCUS devices and the presence of one supervisor per site able to provide ad hoc assistance if needed. We proposed to fellows undergoing their clinical rotations the goal of autonomously performing three or more renal POCUS assessments per week for the following four month period, aiming for a total of 50 studies. During their clinical rotations, nephrology fellows are usually involved in the care of inpatients and ambulatory patients including patients with end-stage kidney disease undergoing maintenance dialysis. They were asked to keep a log to determine how many assessments were performed and were also encouraged to digitally record their assessment for retrospective feedback.

Twenty trainees participated in phase 1. The trainees were generally satisfied with the training session as an introduction to POCUS. Participants emphasized the benefit of having models comprised of both healthy volunteers and volunteers with renal pathologies. Phase 2 of the pilot program was initiated two months after the hands-on training session. The delays were due to obtaining dedicated ultrasound equipment to each site. Fifteen trainees were in clinical rotations during this period. During the first month, it became apparent that the dedicated US devices were underused. The US device was used autonomously 11 times (out of 100 total expected uses) within the first month of phase 2. Furthermore, almost all exams were performed under direct supervision of the local POCUS supervisor. To improve the use of the US device and to stimulate interest, reminders accompanied with ultrasound images and tips were sent to the fellows during the second month of phase 2. However, the US device remained underused during this period. After the second month, phase 2 was interrupted.

This is the first report describing the implementation of a pilot POCUS training program for nephrology fellows in a Canadian institution. The program was based on a structured training session followed by self-directed learning during clinical rotations. The main objective was to provide adequate exposure in an environment where POCUS expertise is scarce. Unfortunately, the program failed to provide adequate exposure to POCUS for nephrology fellows. Despite the success of phase 1, on-site dedicated US devices were underutilized in phase 2 of the project. 

Formal POCUS training is not currently part of the nephrology core curriculum in Canadian institutions. We observed a definitive interest among nephrology fellows to integrate POCUS into their training. An effective training model would require giving basic POCUS training followed by direct hands-on experience. The best method for the latter would be direct supervision by front-line clinicians during clinical rotations 5, although this would require supervisors to be trained in POCUS which represent a significant barrier.

Ensuring adequate POCUS exposure could be achieved through a dedicated ultrasound rotation with an experienced trainer 3. However, learning to integrate POCUS into the clinical decision-making process is an important skill that may be neglected if POCUS is done separately from clinical work. Another solution would be to favor a self-directed learning approach during clinical rotations with rapid access to a local POCUS expert to offer ad hoc supervision and feedback. This approach would have the potential advantage of requiring only a few local POCUS experts while ensuring adequate exposure integrated to clinical work for the fellows in training. However, our experience questions the feasibility of this type of approach.

In summary, this pilot POCUS program based on self-directed learning during clinical rotations was insufficient to provide nephrology trainees with adequate POCUS exposure during fellowship training. However, we identified a strong interest in POCUS among nephrology fellows. During this pilot program, we identified 3 essential components that were missing to ensure adequate exposure to POCUS within the nephrology curriculum: 1) direct supervision, 2) adequate time, and 3) program endorsement of POCUS as an official training objective. Future efforts should focus on providing these essential components to build an effective POCUS program. 

## Statement of Ethics

This is a program report and does not constitute research. No identifiable or sensitive personal information was collected or reported. As such, Research Ethics Board approval was not necessary for the publication of this report. 

## Disclosures

The authors report no financial or other competing interests associated with the present report.
